# Acute Myocardial Infarction Mortality in the Older Population of the United States: An Analysis of Demographic and Regional Trends and Disparities from 1999 to 2022

**DOI:** 10.3390/jcm14072190

**Published:** 2025-03-23

**Authors:** Ali Bin Abdul Jabbar, Mason Klisares, Kyle Gilkeson, Ahmed Aboeata

**Affiliations:** 1 Department of Medicine, Division of Internal Medicine, Creighton University School of Medicine, Omaha, NE 68124, USA; 2 Department of Medicine, Division of Cardiovascular Disease, Creighton University School of Medicine, Omaha, NE 68124, USA

**Keywords:** acute myocardial infarction, mortality, United States, disparities

## Abstract

**Background/Objectives**: Acute myocardial infarction (AMI) has been a leading cause of mortality in the US. Though AMI mortality has been decreasing in the US, significant disparities have persisted. We aim to evaluate disparities in AMI-related deaths in the US from 1999 to 2022. **Methods**: Data from the Centers for Disease Control and Prevention Wide-ranging Online Data for Epidemiologic Research (CDC WONDER) multiple causes of death database were used to analyze death certificates from 1999 to 2022 for AMI-related deaths among United States older adults (aged ≥ 65) for overall trend and disparities based on demographic (sex, race/ethnicity, and ten-year age groups) and regional (census regions, rural-urban status, and states) subgroups. Rural and urban status were distinguished using definitions set by the 2013 NCHS Urban-Rural Classification scheme for counties. These data come from the 2010 Census report and are updated from the 2006 NCHS Urban-Rural Classification scheme for counties. The crude mortality rate (CMR) and age-adjusted mortality rates (AAMRs) per 100,000 people were used to calculate annual percentage changes (APCs) and average annual percentage changes (AAPCs) using Joinpoint regression analysis. **Results**: From 1999 to 2022, there were 3,249,542 deaths due to AMI. Overall, age-adjusted mortality rates (AAMRs) decreased by 62.78% from 563.2 * (95% CI 560.3–565.7) in 1999 to a nadir at 209.6 * (208.3–210.8) in 2019, with an AAPC of −4.96 * (95% CI −5.11 to −4.81). There were a total of 355,441 deaths from AMI from 2020 to 2022; 21,216 (5.97%) of those were from AMI with COVID-19 infection. An increase of 11.4% was observed from an AAMR of 209.6 * (95% CI 208.3–210.8) in 2019 to 233.5 * (95% CI 232.2–234.8) in 2021. From 2021 to 2022, the AAMR of AMI decreased from 233.5 * (95% CI 232.2–234.8) to 209.8 * (95% CI 208.6–211), recovering to the 2019 levels. The AAMR for AMI excluding associated COVID-19 infection was 217.2 at its peak in 2021, which correlates to only a 3.63% increase from 2019. Significant disparities in AMI mortality were observed, with higher mortality rates in men, African Americans, the oldest age group (age ≥ 85), and those living in southern states and rural areas. **Conclusions**: AMI mortality in the older adult population of the US has significantly decreased from 1999 to 2019, with a brief increase during the pandemic from 2019 to 2021, followed by recovery back to the 2019 level in 2022. The majority of the rise observed during the pandemic was associated with COVID-19 infection. Despite remarkable improvement in mortality, significant disparities have persisted, with men, African Americans, and those living in rural areas and the southern region of the US having disproportionately higher mortality.

## 1. Introduction

Acute myocardial infarction (AMI) is a leading cause of cardiovascular and overall mortality in the elderly population of the US. However, myocardial infarction mortality has declined over the past couple of decades [1]. There have been extensive advancements in the care of AMI, including improvements in pharmacological therapeutics such as newer antiplatelets, anticoagulants, statins, and the early recognition and treatment of AMI with percutaneous coronary intervention (PCI) and/or coronary artery bypass grafting (CABG) with more elaborate guidelines to guide management. Moreover, the early recognition and implementation of primary and secondary prevention strategies in managing coronary artery disease (CAD) have also contributed to beneficial trends in mortality and outcomes associated with AMI [2]. Despite the declining trends, the US’s aging ‘baby boomer’ population is adding to the burden CAD has on the healthcare system. The population of those 65 years and older is projected to be nearly 80 million by 2040 [3]. Given the aging population, it is essential to understand the trends of AMI mortality in the older adult population of the US and assess for disparities that may be prevalent to target disadvantaged groups. Understanding demographic and regional differences in mortality can help improve preventive strategies targeting those who have higher mortality rates. Therefore, we aim to analyze trends of AMI-related mortality in the older adult population of the US from 1999 to 2022.

## 2. Methods

### 2.1. Study Design and Database

The Centers for Disease Control and Prevention Wide-ranging Online Data for Epidemiologic Research (CDC WONDER) were used to identify AMI mortality in the older adult population of the US, which was taken as age ≥65 years to focus on the growing population of this age group. The Multiple Cause-of-Death Public Use Records of death certificates were used to extract AMI as an underlying (primary) or contributing cause on nationwide death certificate records [4,5]. This database has been extensively used to study trends and disparities in mortality from various cardiovascular conditions in the US [6] (pp. 1999−2022). This study was exempt from institutional review board approval because the CDC WONDER database contains anonymized, publicly available data. Data for AMI mortality was extracted from 1999 to 2022 using ICD 10 Clinical Modification codes I21.0, I21.1, I21.2, I21.3, I21.9, I22.0, I22.1, I22.8, and I22.9 [7].

### 2.2. Study Population

The analysis included demographic (sex, race/ethnicity, and age groups) and geographic (region, state, and urban-rural classification) categories. The CDC WONDER categorizes gender as male or female based on death certificate information. Race/ethnicity was categorized into non-Hispanic (NH) White, NH Black or African American, NH Asian or Pacific Islander, NH American Indian or Alaska Native, and Hispanic or Latino. According to Census Bureau definitions, regions were classified as Northeast, Midwest, South, and West. Age groups were divided into 65–74, 75–84, and 85 and older. For urban-rural classifications, the National Center for Health Statistics Urban-Rural Classification Scheme was used to divide the population into urban, which included large metropolitan areas (population  ≥  1 million) and medium/small metropolitan areas (population 50,000 to 999,999), and rural (population  <  50,000) counties per the 2013 United States census classification [8].

### 2.3. Statistical Analysis

AMI age-adjusted mortality rates (AAMRs) per 100,000 were calculated. AAMR considers the population’s age distribution variation, enabling better data comparison. The study used the Joinpoint Regression Program (Joinpoint version 5.3.0) available from the National Cancer Institute, Bethesda, Maryland) to analyze mortality trends from 1999 to 2019 [9]. This program identifies significant changes in annual mortality trends by fitting models of linear segments. The annual percentage changes (APCs) with 95% confidence intervals (CIs) for the age-adjusted mortality rates (AAMRs) were calculated for the line segments connecting a Joinpoint. The weighted average of the APCs was calculated and reported as average annual percentage changes (AAPCs), along with corresponding 95% CIs, to summarize the reported mortality trend for the entire study period. APCs and AAPCs were identified as increasing or decreasing based on whether the change in mortality over the time interval significantly differed from zero using a 2-tailed *t*-test. Statistical significance was set at *p*  ≤  0.05 (represented by an asterisk, ‘*’, in results and figures).

The years from 2020 to 2022 were not used in the Joinpoint analysis to prevent the excess mortality during the COVID-19 pandemic from impacting the long-term trend analysis. Instead, the percentage change (PC) in AAMRs from 2019 (considered pre-pandemic) to 2021 (considered peak of pandemic years for AMI mortality) was calculated to compare various subgroups and compared with the PC for 1999 to 2019 (Table 1). From 2020 to 2022, deaths due to AMI and COVID-19 (AMI + COVID-19) combined were also calculated to consider the excess AMI-related deaths due to associated COVID-19 infection. These deaths were excluded from calculating AMI-related deaths without associated COVID-19 (AMI—COVID-19) for the years 2020 to 2022. AMI + COVID-19 deaths were found by extracting death certificate records which mentioned both AMI and COVID-19 (ICD-10 code U07.1) as an underlying or contributing cause of death. Sensitivity analysis was performed for overall AMI deaths and AAMRs using the underlying cause of death database.

## 3. Results

**Figure 1:**
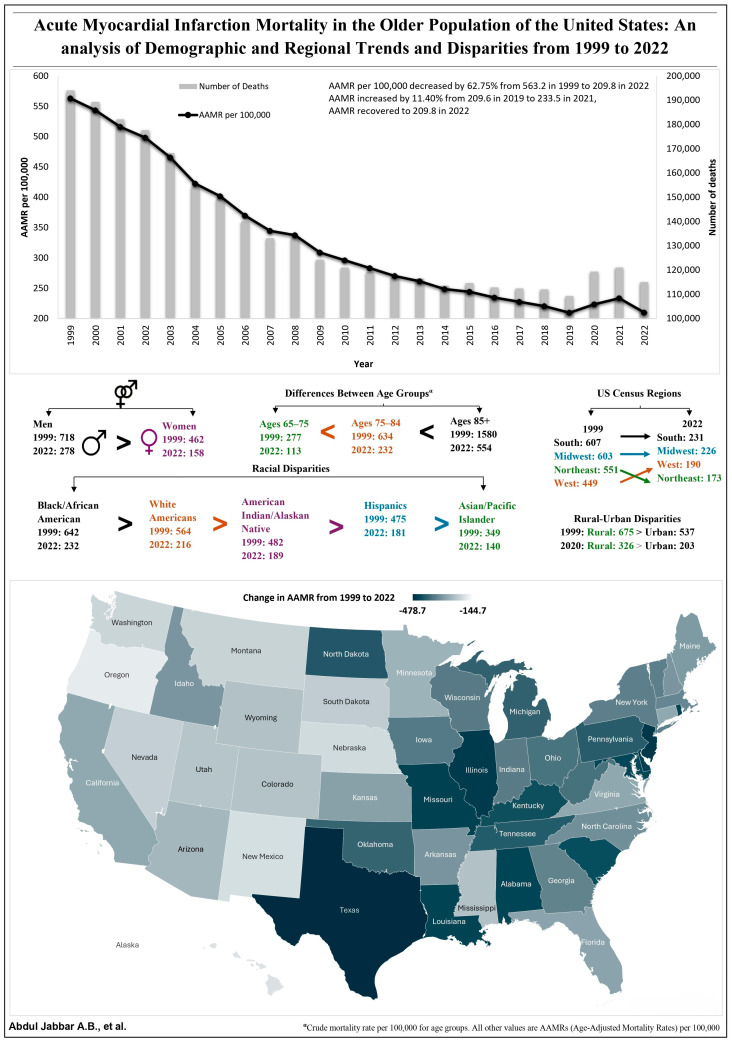
Illustration summarizing the key findings of our study.

**Figure 2:**
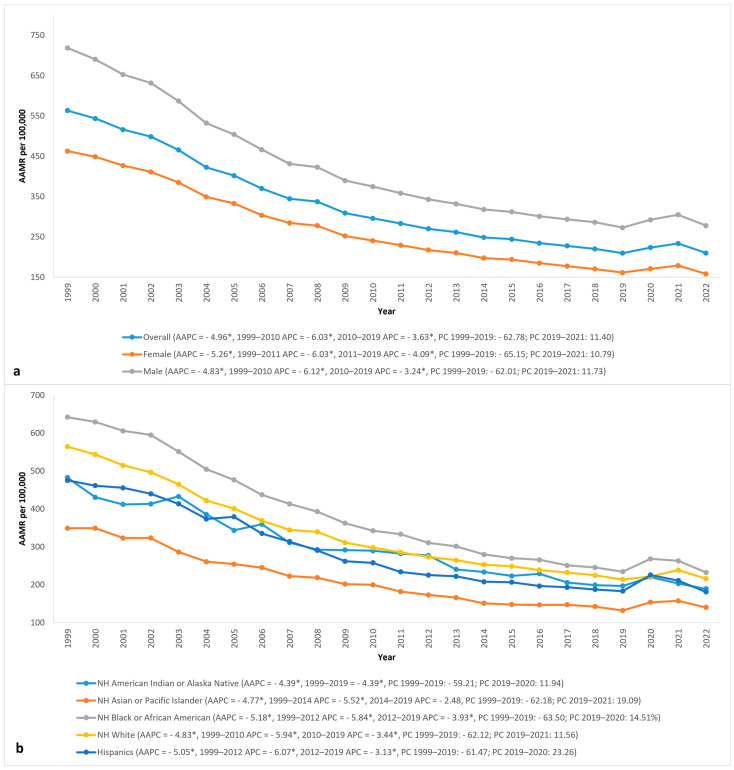
Acute myocardial infarction (AMI) AAMR per 100,000 in the United States, 1999 to 2022: (**a**) overall and stratified by gender; (**b**) stratified by race/ethnicity. APC = annual percent change, * = significantly different from 0 with *p* < 0.05.

**Figure 3:**
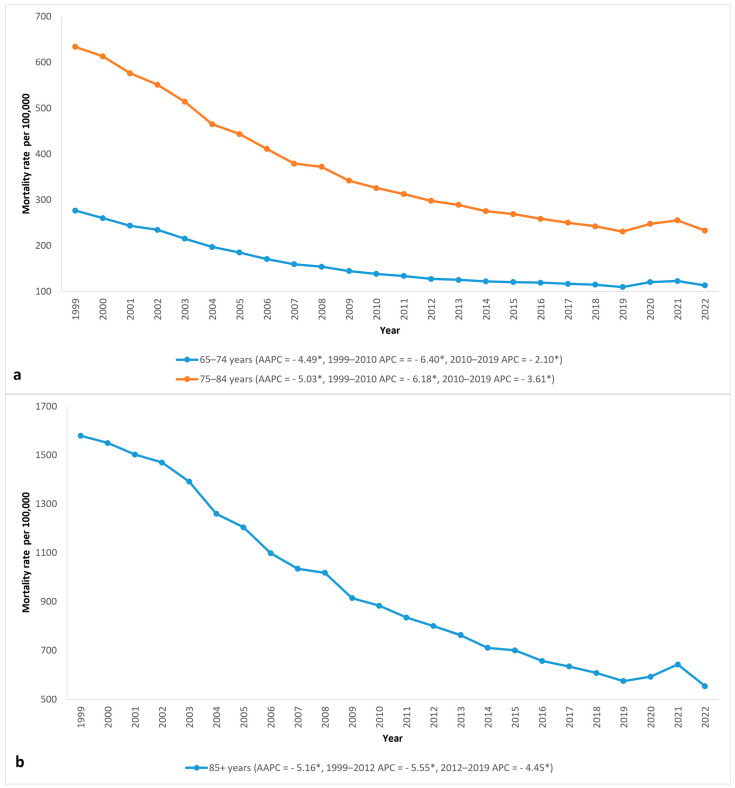
Acute myocardial infarction (AMI) AAMR per 100,000 in the United States, 1999 to 2022, stratified by age groups: (**a**) ages 65–74 and 75–84; (**b**) age ≥ 85. APC = annual percent change, * = significantly different from 0 with *p* < 0.05.

**Figure 4:**
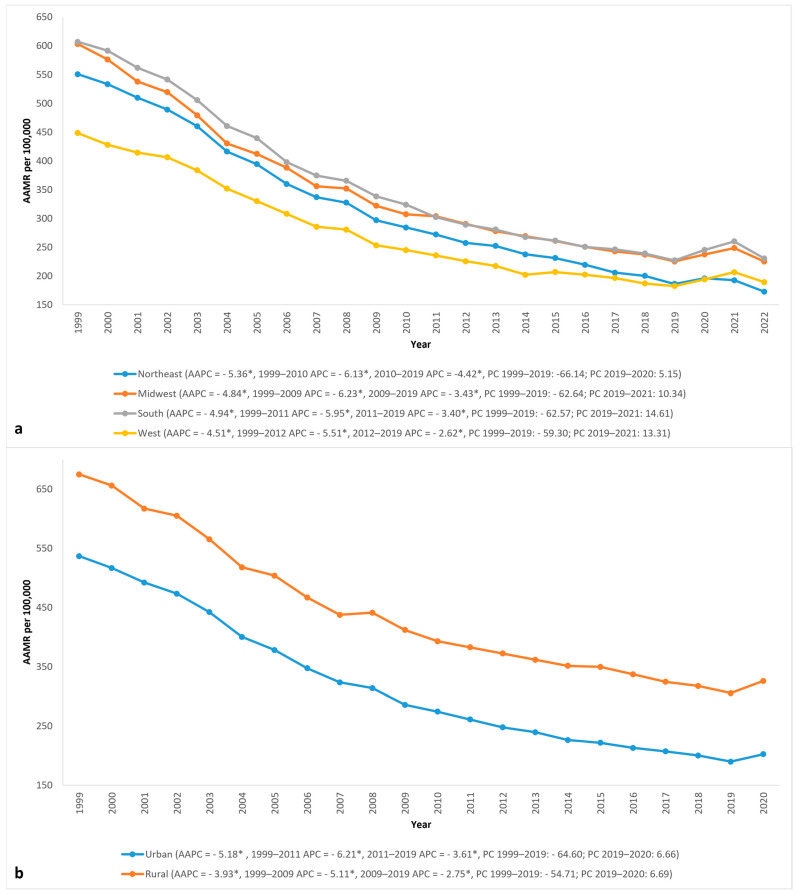
Acute myocardial infarction (AMI) AAMR per 100,000 in the United States, 1999 to 2022, stratified by (**a**) US census regions and (**b**) rural-urban classification. APC = annual percent change, * = significantly different from 0 with *p* < 0.05.

**Figure 5:**
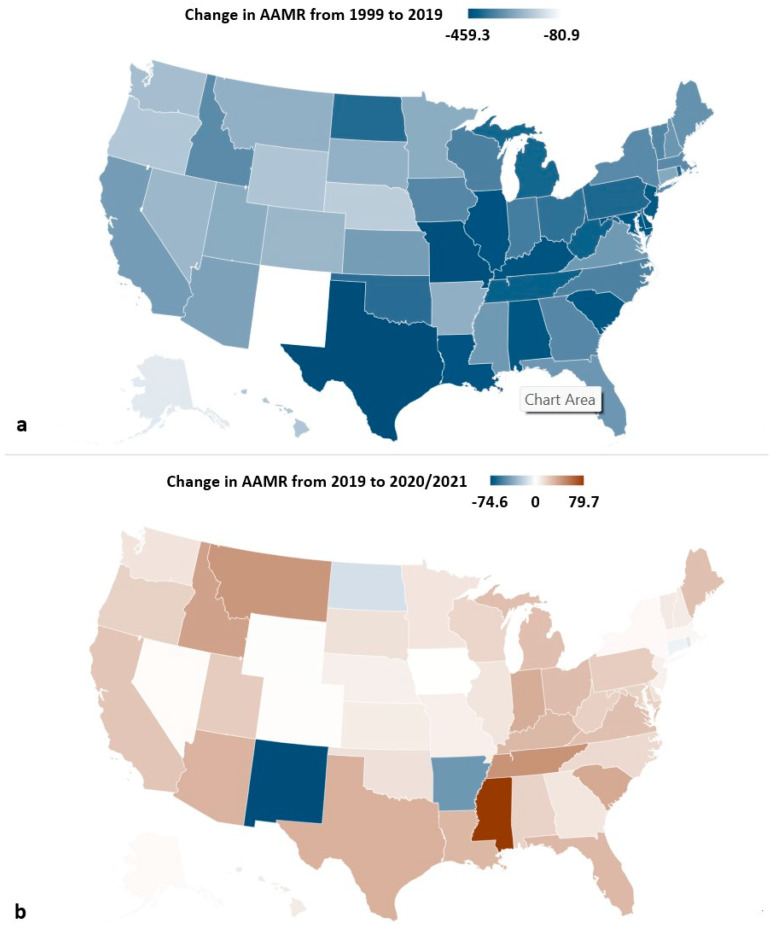
State-level change in acute myocardial infarction (AMI) AAMR per 100,000 in the United States (**a**) from 1999 to 2019 and (**b**) from 2019 to 2020–2021.

Summarized in Figure 1, Figure 2, Figure 3, Figure 4 and Figure 5 and Table 1; the Supplemental File has supporting data.

### 3.1. Overall

#### 3.1.1. Pre-Pandemic Trend (1999 to 2019)

From 1999 to 2022, there were 3,249,542 deaths due to AMI in the United States (Supplemental Table S1). Overall, AAMRs per 100,000 decreased by 62.78% from 563.2 (95% CI 560.3–565.7) in 1999 to a nadir at 209.6 (208.3–210.8) in 2019, with an AAPC of −4.96 * (95% CI −5.11 to −4.81). Sensitivity analysis using AMI as the underlying cause of death revealed a similar trend, with AAMRs decreasing by 68.02% from 474.6 in 1999 to 151.8 in 2019 (Figure 1 and Table 1).

#### 3.1.2. Trends During COVID-19 Pandemic (2020–2022)

There were a total of 355,441 deaths from AMI from 2020 to 2022, and 21,216 (5.97%) of those were from AMI + COVID-19. From 2019 to 2021, there was an 11.4% increase in AAMR from 209.6 (95% CI 208.3–210.8) to 233.5 (95% CI 232.2–234.8), respectively. From 2021 to 2022, the AAMRs of AMI decreased from 233.5 (95% CI 232.2–234.8) to 209.8 (95% CI 208.6–211), which were similar to those seen in 2019, before the pandemic. The AAMR of AMI excluding COVID-19 was 217.2 at its peak in 2021, which correlates to only a 3.63% increase from 2019. Sensitivity analysis using AMI as the underlying cause of death also revealed a 3.82% increase similar to that seen in the AAMR of AMI-COVID-19 in 2021 from AMI in 2019 (Figure 1 and Table 1).

### 3.2. Demographic Disparities

#### 3.2.1. Sex-Stratified Disparities

Men had higher AAMRs than women throughout the study period and saw a greater increase in AMI mortality from 2019 to 2021. From 1999 to 2022, AMI caused 1,604,995 (49.39%) deaths in women and 1,644,547 (50.61%) deaths in men in the United States. In men, the AAMR decreased by 62.01% from 718.4 (95% CI 713.7–723.1) in 1999 to 272.9 (95% CI 270.7–275.1) in 2019 (AAPC: −4.83 *; 95% CI −4.99 to −4.68). The AAMR increased by 11.73% from 272.9 (95% CI 270.7–275.1) in 2019 to 304.9 (95% CI 302.5–307.2), followed by a decrease to 278 (95% CI 275.9–280.2) in 2022. Compared to that, women had a 65.15% decrease in AAMR from 462.8 (95% CI 460–465.7) in 1999 to 161.3 (95% CI 159.8–162.7) in 2019 (AAPC: −5.26 *; 95% CI −5.44 to −5.09). From 2019 to 2021, their AAMR increased by 10.79% from 161.3 (95% CI 159.8–162.7) to 178.7 (95% CI 177.1–180.2), followed by a decrease in 2022 to 158.3 (95% CI 157–159.7) (Figure 2 and Table 1).

#### 3.2.2. Race-Stratified Disparities

Though NH Black or African American people had the highest AAMRs throughout the study, they had the most decrease in AAMR. In contrast, NH Asian or Pacific Islanders consistently had the lowest AAMR from 1999 to 2022. The magnitude of racial differences in AAMR gradually improved over the past 2 decades, though they still exist to a lesser degree. Hispanics saw the highest increase in AAMR during the COVID-19 pandemic. In NH Black or African American people, the AAMRs decreased by 63.5% from 642 (95% CI 632.4–651.6) in 1999 to 234.3 (95% CI 229.8–238.8) in 2019 with an AAPC of −5.18 * (95% CI −5.46 to −4.92). In 2020, the AAMR increased to 268.3 (95% CI 263.6–273), rising 12.67% from 2019, before decreasing to pre-pandemic levels in 2022 (Figure 2 and Table 1).

NH White people had the second highest AAMR, followed by NH American Indian or Alaska Native, Hispanics, and NH Asian or Pacific Islander people. NH Asian or Pacific Islander people had the lowest AAMR throughout the study, with a decrease of 62.18% from 349 (95% CI 334.4 to 363.6) in 1999 to 132 (95% CI 127.4 to 136.6) in 2019, with an AAPC of −4.39 * (95% CI −4.82 to −3.97). Hispanics experienced the greatest increase in AAMR during the COVID pandemic, with an AAMR of 18.87% from 2019 to a peak in 2020, followed by a return to pre-pandemic levels by 2022 (Figure 2 and Table 1).

#### 3.2.3. Age Group Disparities

All 10-year age groups had a decrease in mortality rate from 1999 to 2019, followed by a brief increase from 2019 to 2021 and a return to the 2019 level by 2022. People over 85 had the highest mortality rate of all 10-year age groups but also saw the most improvement in AAMR, which decreased by 64.9% from 1579.6 in 1999 to 554.3 in 2022 (Figure 3 and Table 1).

### 3.3. Regional Differences

#### 3.3.1. Census Region-Based Disparities

Every census region had a decrease in AAMR from 1999 to 2022, similar to the overall trend. The South had the highest AAMR during the initial years, with an AAMR of 607 (95% CI 602.6 to 611.4) in 1999, which decreased to 227.2 (95% CI 225.1 to 229.4) by 2019, which was similar to that of the Midwest at 225.4 (95% CI 222.6–228.2) in 2019. The South also had the highest increase in AAMR from 2019 to 2021, increasing by 14.6% to 260.4 (95% CI 258.1 to 262.7) in 2021, followed by a decrease to 230.8 (95% CI 228.7 to 232.9) by 2022. This general trend was followed by the Midwest, West, and Northeast, with the Northeast witnessing the greatest decrease and achieving the lowest AAMR of all regions in 2022 at 173 (95% CI 170.4 to 175.5) (Figure 4 and Table 1).

#### 3.3.2. Rural vs. Urban Disparities

In rural areas, AAMRs were consistently higher throughout the study than those in urban areas, with less improvement observed. The AAMR in rural areas in 1999 was 674.8 (95% CI 668.5 to 681), which decreased by 54.7% to 305.6 * (95% CI 301.9 to 309.2) in 2019, with an AAPC of −3.93 * (95% CI −4.11 to −3.77). Compared to that, the AAMR of urban areas decreased by 64.6% from 536.7 (95% CI 534–539.4) in 1999 to 190 (95% CI 188.6–191.3) in 2019 (AAPC −5.18 *; 95% CI −5.35 to −5.01). The AAMR in rural and urban areas saw a similar increase of 6.69% and 6.66% from 2019 to 2020 (Figure 4 and Table 1).

### 3.4. State-Level Difference

All states saw a decrease in AAMR from 1999 to 2022, with Texas having the highest AAMR reduction (−478.7) from 718.5 in 1999 to 239.8 in 2022. All states saw a decrease in AAMR from 1999 to 2019, with Texas, Missouri, Illinois, Delaware, and Kentucky having the most significant reductions. From 2019 to 2020–2021, Mississippi, Tennessee, Montana, Idaho, and South Carolina had the greatest increase in AAMR, while New Mexico, Arkansas, North Dakota, Rhode Island, and Connecticut also saw decreases in AAMR during the pandemic (Figure 1 and Figure 5 and Table 1).

## 4. Discussion

Our study sheds light on significant disparities in AMI mortality in the older population of the US. Overall, AAMR decreased to the lowest level in 2019 and briefly saw a mild increase during the pandemic, followed by a decrease in 2022 to levels similar to pre-pandemic levels. In most population subgroups, the decreasing trend of mortality was noted to have halted in 2008, likely due to the economic downturn during the 2008 financial crisis, increasing delays in seeking acute care [10]. We also see that most of the rise during the pandemic was attributed to excess AMI mortality with an overlap of COVID-19 infection, with around 3.6% increase contributing to AMI deaths without COVID-19 infection. Men had higher mortality throughout and saw a more significant jump in mortality during the pandemic. NH African Americans had the highest AAMR throughout the study, whereas Hispanics saw the greatest increase during the pandemic. South and Midwest regions had the highest AAMR before the pandemic, but the South witnessed the highest growth, leading to higher AAMR than the Midwest during the pandemic.

Multiple factors have contributed to the decrease in mortality, such as emergency medical services (EMSs) performing prehospital EKGs, the statewide adoption of STEMI hospital destination policies allowing EMSs to bypass non-PCI-capable facilities, the shortened time to perfusion with PCI, and the increased use of primary PCI [11,12] medications. This, along with the increased use of guideline-based treatments, better management of long-term comorbidities, and better post-AMI and revascularization care, contributed to an overall decrease in AMI mortality.

The mild increase in AAMR due to the COVID-19 pandemic was primarily because of excess deaths driven directly by COVID-19 infection and delays in seeking healthcare. The pandemic’s dramatic impact on the healthcare system, overcrowded emergency departments, individual decisions to avoid medical facilities to avoid COVID-19 exposure, and financial concerns led to delayed presentation and intervention for AMI [13]. Additionally, patients may have avoided outpatient visits more, leading to ineffective monitoring of risk factors for AMI. The pandemic also changed the lifestyle of much of the world, as there was a rise in sedentary lifestyles and an increase in working from home, which would impact cardiovascular health beyond the pandemic. COVID-19 infection has been found to cause systemic inflammation, causing endothelial activation, hypercoagulability, and arterial and venous thrombosis, thus contributing directly to an increase in AMI mortality, as seen in our analysis [14,15]. Additionally, 10–20% of patients who had a COVID-19 infection in hospital experienced evidence of myocardial injury thought to be caused by increased myocardial stress and demand from the cytokine surge in acute illness, myocarditis, strain from acute respiratory distress syndrome, and increased pulmonary arterial pressures [15].

Men consistently had a higher AAMR every year, likely due to the hormonal differences favoring women, which is well known to protect women by slowing the rate of development of atherosclerotic cardiovascular disease (ASCVD) compared to males [16]. In addition, men generally have a higher prevalence of traditional risk factors such as dyslipidemia, hypertension, smoking, and alcohol consumption, which contribute to a higher incidence of AMI [17,18].

Cardiovascular disease mortality, including AMI Americans, has been worse for NH Black or Africans of all racial and ethnic groups, with multiple factors to blame. Social determinants of health include increased poverty, lower education levels, and low health literacy, leading to higher rates of risk factors for ASCVD, such as hypertension, underdiagnosed T2DM, obesity, poorer diet quality, decreased physical activity, and poor sleep quality, which have been well documented in prior studies [19]. The range between the highest and lowest AAMR amongst all races from the beginning to the end of the study decreased, indicating a narrowing of racial disparities. This is likely due to Medicaid expansion under the Affordable Care Act, the federal initiative to eliminate racial and ethnic disparities in health, which was launched in 1998, aiming to improve racial disparities [20,21]. In addition, recent pushes of awareness campaigns by societies such as the American College of Cardiology and other institutions towards addressing social determinants of health, with aims to increase screening for risk factors like transportation barriers and financial strain, helped physicians address these issues and tailor care plans [22].

An increase in age brings an increased morbidity burden, which, in turn, increases the risk of mortality from AMI. Moreover, older adults are more likely to have contraindications to reperfusion eligibility, and even when eligible for reperfusion therapy, they are less likely to receive it [23]. Further complicating AMI presentation, elderly patients are more likely to exhibit atypical features, which complicate the timely identification of AMI and lead to delayed treatment [24]. Despite this, the most dramatic decrease in mortality amongst older patients occurred in the 85+ age group. More significant improvements in the mortality rate likely occurred because this age group had the highest AAMR at the start of the study, thus creating greater room for improvement.

Of the four census regions, the South had the highest mortality for most years. The difference in AAMR narrowed during the later years, with the South and Midwest having similar AAMR in some of the last years. Cardiovascular risk factors such as obesity, hypertension, smoking, and diabetes remain more prevalent in the South, driven by poverty and lack of awareness due to lower levels of healthcare literacy and healthcare access, keeping AMI mortality higher than in other regions [25]. States that had the greatest increase in AMI mortality during the pandemic were also concentrated in the South, such as Mississippi and Tennessee. Interestingly, despite being in the South, Arkansas saw one of the most significant decreases in AMI mortality during the pandemic. Lower mortality rates in urban areas have primarily been linked to shorter travel distances for emergency care, timely accessibility, and transfer to PCI/CABG-capable hospitals, leading to higher rates of interventional treatment [26]. When comparing rural vs. urban areas, the rate of cardiac catheterization within 30 days of AMI is 49.7% vs. 63.6%, and for PCI, it is 42.1% vs. 45.7% [27]. Rural areas experience longer 911 call-to-hospital arrival times and extended transport times if a transfer to a capable facility is warranted since there is limited availability of advanced cardiac services compared to urban areas [26,28]. Along with that, better access to preventive care for ASCVD has contributed to lower rates of smoking, obesity, and hypertension, thus leading to persistently lower AMI incidence and mortality rates in urban populations [29].

## 5. Limitations

Data from CDC WONDER are derived from death certificates, which utilize ICD codes for identifying diseases, thereby introducing the possibilities of misclassification bias and underreporting or inconsistencies in cause-of-death coding across regions. A rural-urban stratified analysis could only be performed from 1999 to 2020, as the database does not provide age group-specific population figures or mortality rates for rural and urban areas beyond 2020. Additionally, due to the nature of the data, it is not possible to establish a causal connection between AMI and COVID-19 infection for the deaths during the pandemic or any of the AMI risk factors for the duration of the study. Further limitations include the unavailability of adherence to drug therapy and participation in cardiac rehabilitation programs in the database.

## 6. Conclusions

AMI mortality in the older adult population of the US significantly decreased from 1999 to 2019, with a brief increase during the pandemic from 2019 to 2021, followed by recovery back to the 2019 level in 2022. The majority of the rise observed during the pandemic was associated with COVID-19 infection. Despite remarkable improvement in mortality, significant disparities have persisted, with men, African Americans, and those living in rural areas and the southern region of the US having disproportionately higher mortality.

## Figures and Tables

**Table 1:** Summary of change in AAMRs from 1999 to 2019 and the increase from 2019 to 2020/2021 (most subgroups had a pandemic peak in 2021; ** represents the groups with a peak in 2020 and thus those that were used to calculate percentage increase during the pandemic; * = significantly different AAPC from 0 with *p* < 0.05).

	Crude Number of Deaths (1999)	AAMR 1999 (95% CI)	Crude Number of Deaths (2019)	AAMR 2019 (95% CI)	AAPC from 1999 to 2019 (95% CI)	Crude Number of Deaths (2021)	AAMR 2021 (95% CI)	Percentage Change from 1999 to 2019 (95% CI) (%)	Percentage Change from 2019 to 2020/2021 (95% CI) (%)
Overall	194,044	563.2 (560.3–565.7)	109,282	209.6 (208.3210.8)	−4.96 * (−5.11 to −4.81)	121,052	233.5 (232.2–234.8)	−62.78 (−62.82 to −62.74)	11.40 (11.37 to 11.43)
**Sex-stratified**									
Male	93,111	718.4 (713.7–723.1)	60,234	272.9 (270.7–275.1)	−4.83 * (−4.99 to −4.68)	68,155	304.9 (302.5–307.2)	−62.01 (−62.07 to −61.96)	11.73 (11.70 to 11.76)
Female	100,933	462.8 (460–465.7)	49,048	161.3 (159.8–162.7)	−5.26 * (−5.44 to −5.09)	52,897	178.7 (177.1–180.2)	−65.15 (−65.26 to −65.06)	10.79 (10.76 to 10.82)
**Race-Stratified**									
NH American Indian or Alaska Native	569	482.5 (441.9–523)	590	196.8 (180.5–213.1)	−4.39 * (−4.82 to −3.97)	618	203.1 ** (186.6–219.6)	−59.21 (−59.15 to −59.25)	11.94 (11.91 to 11.97)
NH Asian or Pacific Islander	2332	349 (334.4–363.6)	3217	132 (127.4–136.6)	−4.77 * (−5.10 to −4.49)	3978	157.2 (152.3–162.2)	−62.18 (−61.90 to −62.43)	19.09 (19.05 to 19.13)
NH Black or African American	17,220	642 (632.4–651.6)	10,883	234.3 (229.8–238.8)	−5.18 * (−5.46 to −4.92)	12,501	262.9 ** (258.1–267.6)	−63.50 (−63.66 to −63.35)	14.51 (14.48 to 14.54)
NH White	166,625	564.1 (561.4–566.9)	86,539	213.7 (212.3–215.2)	−4.83 * (−4.98 to −4.68)	93,785	238.4 (236.9–239.9)	−62.12 (−62.18 to −62.04)	11.56 (11.53 to 11.59)
Hispanic	6789	475.2 (463.7–486.8)	7854	183.1 (179–187.2)	−5.05 * (−5.36 to −4.75)	9553	210.9 ** (206.6–215.2)	−61.47 (−61.40 to −61.54)	23.27 (23.23 to 23.31)
**Census Region**									
Northeast	40,785	550.8 (545.5–556.2)	18,318	186.5 (183.8–189.2)	−5.36 * (−5.64 to −5.13)	18,834	192.8 ** (190–195.6)	−66.14 (−66.31 to −65.98)	5.15 (5.12 to 5.18)
Midwest	50,271	603.3 (598–608.5)	25,427	225.4 (222.6–228.2)	−4.84 * (−4.98 to −4.71)	18,834	248.7 (245.8–251.7)	−62.64 (−62.78 to −62.50)	10.34 (10.31 to 10.37)
South	50,271	607 (602.6–611.4)	25,427	227.2 (225.1–229.4)	−4.94 * (−5.12 to −4.78)	50,945	260.4 (258.1–262.7)	−62.57 (−62.65 to −62.48)	14.61 (14.58 to 14.64)
West	30,050	448.6 (443.5–453.7)	21,085	182.6 (180.1–185.1)	−4.51 * (−4.74 to −4.28)	23,717	206.9 (204.2–209.5)	−59.30 (−59.39 to −59.20)	13.31 (13.28 to 13.34)
**Urbanization Status**						**2020**			
Rural	45,091	674.8 (668.5–681)	27,060	305.6 (301.9–309.2)	−3.93 * (−4.11 to −3.77)	29,405	326.03 (322.8–329.78)	−54.71 (−54.84 to −54.60)	6.69 (6.66 to 6.72)
Urban	148,953	536.7 (534–539.4)	82,222	190 (188.6–191.3)	−5.18 * (−5.35 to −5.01)	89,968	202.65 (201.31–203.98)	−64.60 (−64.68 to −64.53)	6.66 (6.63 to 6.69)

## Data Availability

The data presented in this study are publicly available through the CDC WONDER website: https://wonder.cdc.gov/.

## Supplementary Materials

The following supporting information can be downloaded at: https://www.mdpi.com/article/10.3390/jcm14072190/s1, Figure S1. APC for overall and gender stratified AMI-related mortality rate in the older adult population of US United States from 1999 to 2019. * Indicates the APC is significantly different from 0; Figure S2. APC for Race stratified AMI-related mortality rate in the older adult population of US United States from 1999 to 2019. * Indicates the APC is significantly different from 0; Figure S3. APC for age-stratified AMI-related mortality rate in the older adult population of the United States from 1999 to 2019. * Indicates the APC is significantly different from 0; Figure S4. APC for census region-stratified AMI-related mortality rate in the older adult population of the United States from 1999 to 2019. * Indicates the APC is significantly different from 0; Figure S5. APC for urban-rural-stratified AMI-related mortality rate in the older adult population of the United States from 1999 to 2019. * Indicates the APC is significantly different from 0; Figure S6. Sensitivity analysis and AMI deaths without COVID-19; Figure S7. State-level change in AMI-related AAMR from 1999 to 2019; Figure S8. State-level change in AMI-related AAMR from 2019 to 2020-2021 period; Table S1. AMI-related crude number of deaths in the older adult population of the US stratified by sex, race, region, age group, Census region and urbanization status in the United States; Table S2. AMI-related mortality rates stratified by sex, race, and age in the United States; Table S3. AMI-related mortality rates are stratified by the census region and urbanization status in the United States; Table S4. AMI-related change in AAMR at state level in United States; Table S5. Sensitivity analysis using AMI as an underlying cause of death, AMI + COVID and AMI − COVID deaths, and AAMR.

## References

[1] Martin S.S., Aday A.W., Almarzooq Z.I., Anderson C.A.M., Arora P., Avery C.L., Baker-Smith C.M., Barone Gibbs B., Beaton A.Z., Boehme A.K. (2024). 2024 Heart Disease and Stroke Statistics: A Report of US and Global Data From the American Heart Association. Circulation.

[2] Abdul Jabbar A.B., Ismayl M., Mishra A., Walters R.W., Goldsweig A.M., Aronow H.D., Tauseef A., Aboeata A.S. (2024). Outcomes of Acute Myocardial Infarction in Patients with Systemic Lupus Erythematosus: A Propensity-Matched Nationwide Analysis. Am. J. Cardiol..

[3] Odden M.C., Coxson P.G., Moran A., Lightwood J.M., Goldman L., Bibbins-Domingo K. (2011). The impact of the aging population on coronary heart disease in the United States. Am. J. Med..

[4] Multiple Cause of Death, 1999–2020 Request. https://wonder.cdc.gov/mcd-icd10.html.

[5] Multiple Cause of Death, 2018–2023, Single Race Request. https://wonder.cdc.gov/mcd-icd10-expanded.html.

[6] Abdul Jabbar A.B., May M.T., Deisz M., Tauseef A. (2025). Trends in heart failure-related mortality among middle-aged adults in the United States from 1999-2022. Curr. Probl. Cardiol..

[7] World Health Organization (2004). ICD-10: International Statistical Classification of Diseases and Related Health Problems: Tenth Revision.

[8] Ingram D.D., Franco S.J. (2014). 2013 NCHS Urban-Rural Classification Scheme for Counties. Vital Health Stat..

[9] Kim H.J., Fay M.P., Feuer E.J., Midthune D.N. (2000). Permutation tests for joinpoint regression with applications to cancer rates. Stat. Med..

[10] Fiuzat M., Shaw L.K., Thomas L., Felker G.M., O’Connor C.M. (2010). United States stock market performance and acute myocardial infarction rates in 2008-2009 (from the Duke Databank for Cardiovascular Disease). Am. J. Cardiol..

[11] Granger C.B., Bates E.R., Jollis J.G., Antman E.M., Nichol G., O’Connor R.E., Gregory T., Roettig M.L., Peng S.A., Ellrodt G. (2019). Improving Care of STEMI in the United States 2008 to 2012. J. Am. Heart Assoc..

[12] Green J.L., Jacobs A.K., Holmes D., Chiswell K., Blanco R., Bates E.R., French W., Kupas D.F., Mears G., Roe M. (2018). Taking the Reins on Systems of Care for ST-Segment-Elevation Myocardial Infarction Patients: A Report from the American Heart Association Mission: Lifeline Program. Circ. Cardiovasc. Interv..

[13] Anderson K.E., McGinty E.E., Presskreischer R., Barry C.L. (2021). Reports of Forgone Medical Care Among US Adults During the Initial Phase of the COVID-19 Pandemic. JAMA Netw. Open.

[14] Giustino G., Pinney S.P., Lala A., Reddy V.Y., Johnston-Cox H.A., Mechanick J.I., Halperin J.L., Fuster V. (2020). Coronavirus and Cardiovascular Disease, Myocardial Injury, and Arrhythmia: JACC Focus Seminar. J. Am. Coll. Cardiol..

[15] Bozkurt B., Das S.R., Addison D., Gupta A., Jneid H., Khan S.S., Koromia G.A., Kulkarni P.A., LaPoint K., Lewis E.F. (2022). 2022 AHA/ACC Key Data Elements and Definitions for Cardiovascular and Noncardiovascular Complications of COVID-19: A Report of the American College of Cardiology/American Heart Association Task Force on Clinical Data Standards. Circ. Cardiovasc. Qual. Outcomes.

[16] Maas A.H.E.M., Appelman Y.E.A. (2010). Gender differences in coronary heart disease. Neth. Heart J. Mon. J. Neth. Soc. Cardiol. Neth. Heart Found..

[17] Madssen E., Laugsand L.E., Wiseth R., Mørkedal B., Platou C., Vatten L., Janszky I. (2013). Risk of acute myocardial infarction: Dyslipidemia more detrimental for men than women. Epidemiol. Camb. Mass.

[18] Albrektsen G., Heuch I., Løchen M.-L., Thelle D.S., Wilsgaard T., Njølstad I., Bønaa K.H. (2016). Lifelong Gender Gap in Risk of Incident Myocardial Infarction: The Tromsø Study. JAMA Intern. Med..

[19] Carnethon M.R., Pu J., Howard G., Albert M.A., Anderson C.A.M., Bertoni A.G., Mujahid M.S., Palaniappan L., Taylor H.A., Willis M. (2017). Cardiovascular Health in African Americans: A Scientific Statement from the American Heart Association. Circulation.

[20] Bhatia N., Vakil D., Zinonos S., Cabrera J., Cosgrove N.M., Dastgiri M., Kostis J.B., Kostis W.J., Moreyra A.E. (2023). Myocardial Infarction Data Acquisition System (MIDAS 44) Study Group * US Initiative to Eliminate Racial and Ethnic Disparities in Health: The Impact on the Outcomes of ST-Segment-Elevation Myocardial Infarction in New Jersey. J. Am. Heart Assoc..

[21] Valdovinos E.M., Niedzwiecki M.J., Guo J., Hsia R.Y. (2020). The association of Medicaid expansion and racial/ethnic inequities in access, treatment, and outcomes for patients with acute myocardial infarction. PLoS ONE.

[22] Virani S.S., Newby L.K., Arnold S.V., Bittner V., Brewer L.C., Demeter S.H., Dixon D.L., Fearon W.F., Hess B., Johnson H.M. (2023). 2023 AHA/ACC/ACCP/ASPC/NLA/PCNA Guideline for the Management of Patients with Chronic Coronary Disease: A Report of the American Heart Association/American College of Cardiology Joint Committee on Clinical Practice Guidelines. Circulation.

[23] Forman D.E., Chen A.Y., Wiviott S.D., Wang T.Y., Magid D.J., Alexander K.P. (2010). Comparison of outcomes in patients aged <75, 75 to 84, and ≥ 85 years with ST-elevation myocardial infarction (from the ACTION Registry-GWTG). Am. J. Cardiol..

[24] Brieger D., Eagle K.A., Goodman S.G., Steg P.G., Budaj A., White K., Montalescot G. (2004). Acute Coronary Syndromes Without Chest Pain, An Underdiagnosed and Undertreated High-Risk Group: Insights from The Global Registry of Acute Coronary Events. Chest.

[25] Senney G.T., Steckel R.H. (2021). Developmental Origins of Cardiovascular Disease: Understanding High Mortality Rates in the American South. Int. J. Environ. Res. Public. Health.

[26] Loccoh E.C., Joynt Maddox K.E., Wang Y., Kazi D.S., Yeh R.W., Wadhera R.K. (2022). Rural-Urban Disparities in Outcomes of Myocardial Infarction, Heart Failure, and Stroke in the United States. J. Am. Coll. Cardiol..

[27] Balamurugan A., Delongchamp R., Im L., Bates J., Mehta J.L. (2016). Neighborhood and Acute Myocardial Infarction Mortality as Related to the Driving Time to Percutaneous Coronary Intervention–Capable Hospital. J. Am. Heart Assoc..

[28] Stopyra J.P., Crowe R.P., Snavely A.C., Supples M.W., Page N., Smith Z., Ashburn N.P., Foley K., Miller C.D., Mahler S.A. (2023). Prehospital Time Disparities for Rural Patients with Suspected STEMI. Prehosp. Emerg. Care.

[29] Liu L., Yin X., Chen M., Jia H., Eisen H.J., Hofman A. (2018). Geographic Variation in Heart Failure Mortality and Its Association with Hypertension, Diabetes, and Behavioral-Related Risk Factors in 1,723 Counties of the United States. Front. Public Health.

