# Supplementary material for: Acute Myocardial Infarction Mortality in the Older Population of the United States: An Analysis of Demographic and Regional Trends and Disparities from 1999 to 2022
Source: J Clin Med. 2025 Mar 23;14(7):2190. doi: 10.3390/jcm14072190 (PMC11989499; doi:10.3390/jcm14072190)

Supplemental Figures and Tables:

Supplemental Table 1. AMI-related crude number of deaths in the older adult population of the US stratified by sex, race, region, age group, Census region and urbanization status in the United States.

Year	Overall	Female	Male	NH American Indian or Alaska Native	NH Asian or Pacific Islander	NH Black or African American	NH White	Hispanics	65-74 years	75-84 years	85+ years	Northeast	Midwest	South	West	Urban	Rural
1999	194044	100933	93111	569	2332	17220	166625	6789	50924	77505	65615	40785	50271	50271	30050	148953	45091
2000	189291	98959	90332	548	2493	17033	161817	6922	47802	75790	65699	39949	48339	48339	29168	145139	44152
2001	182116	95270	86846	535	2500	16599	154787	7266	44738	72573	64805	38580	45559	45559	28916	140374	41742
2002	177669	92463	85206	551	2660	16403	150248	7342	43122	70348	64199	37334	44308	44308	28841	136595	41074
2003	168226	87608	80618	596	2509	15379	142141	7249	39816	66277	62133	35521	41300	41300	27806	129574	38652
2004	154439	80033	74406	544	2421	14275	130016	6896	36770	60417	57252	32304	37392	37392	25972	118800	35639
2005	149410	77378	72032	490	2531	13747	125056	7382	34904	57987	56519	30943	36195	36195	25016	114412	34998
2006	140028	71696	68332	529	2575	12880	116943	6849	32787	53804	53437	28564	34571	34571	23908	107154	32874
2007	133128	68393	64735	479	2481	12434	110854	6736	31409	49607	52112	27149	32210	32210	22765	101894	31234
2008	133113	67913	65200	472	2579	12139	111120	6590	31583	48650	52880	26769	32334	32334	22997	101202	31911
2009	124226	62507	61719	479	2534	11514	103211	6302	30687	44506	49033	24539	29919	29919	21371	94088	30138
2010	121035	60510	60525	506	2591	11100	100171	6449	30017	42542	48476	23794	28926	28926	21119	91936	29099
2011	119111	59074	60037	528	2592	11228	98222	6348	30041	41188	47882	23208	29171	29171	21108	90233	28878
2012	117083	57343	59740	550	2651	10891	96229	6492	30518	39502	47063	22426	28461	28461	20955	88359	28724
2013	116479	56506	59973	513	2756	11014	95139	6805	31544	38859	46076	22379	27816	27816	20826	87973	28506
2014	113582	54110	59472	524	2710	10657	92568	6817	32141	37653	43788	21312	27474	27474	20104	85399	28183
2015	114613	54277	60336	535	2869	10721	92852	7236	33149	37430	44034	21066	27113	27113	21267	86067	28546
2016	112837	52687	60150	578	3000	10985	90713	7238	34081	36815	41941	20279	26398	26398	21469	84849	27988
2017	112402	51727	60675	548	3238	10824	89948	7561	34575	36763	41064	19590	26225	26225	21358	84895	27507
2018	111987	50737	61250	576	3295	11006	89155	7687	34947	37250	39790	19384	26236	26236	21012	84443	27544
2019	109282	49048	60234	590	3217	10883	86539	7854	34512	36804	37966	18318	25427	25427	21085	82222	27060
2020	119373	52805	66568	700	4000	12972	91293	10177	39174	40714	39485	19516	27248	27248	23118	89968	29405
2021	121052	52897	68155	618	3978	12501	93785	9553	41285	41351	38416	18834	27556	50945	23717	-	-
2022	115016	50121	64895	600	3852	11504	89802	8663	38260	40807	35949	17741	26321	47845	23109	-	-

Supplemental Table 2. AMI-related mortality rates stratified by sex, race, and age in the United States.

Year	Overall	Female	Male	NH American Indian or Alaska Native	NH Asian or Pacific Islander	NH Black or African American	NH White	Hispanics	65-74 years	75-84 years	85+ years
1999	563.2	462.8	718.4	482.5	349	642	564.1	475.2	276.5	634	1579.6
2000	543.5	448.6	690.1	430.7	348.9	629.3	543.6	461.2	259.9	613.1	1549.7
2001	516	426.8	652.4	411.8	323.2	605.9	515	455.7	243.4	576.3	1502.7
2002	498.4	411.1	631.6	413.2	323	595	496.5	439.9	234.5	551.1	1469.5
2003	465.5	385	586.7	432.7	286.3	551	464.7	413.3	215.2	513.9	1391.2
2004	422.5	349.1	532	385.3	260.6	504.7	421.9	373.4	197	465.1	1259.4
2005	401.7	332.7	503.9	343.5	254.1	476.5	400.5	379.2	184.9	443.5	1204.2
2006	369.7	303.5	466.3	359.6	245.3	437.4	369.2	335	170.7	410.9	1098.2
2007	344.6	284.7	431.2	310.7	222.7	413	344.5	313.9	159.4	379	1034.1
2008	337.4	277.8	422.7	292.6	218.6	392.7	339.3	290.4	154	372.1	1017.7
2009	308.9	252.2	389.5	291.5	201.8	362.3	310.7	261.9	144.5	341.8	913.6
2010	296	240.6	374.6	289.5	199.7	342.5	297.7	257.7	138.2	325.7	882.4
2011	283.1	229.3	358.3	281.9	181.7	333.5	285.6	233.8	133.6	312.6	834.6
2012	270.1	217.4	342.9	277	173.2	310.5	273.3	225.3	127.2	297.6	799.4
2013	261.5	210.1	331.8	240.4	166.2	301.4	264.6	222.1	125.1	289	762.7
2014	248.5	197.3	317.8	233.6	151.2	279.7	252.8	208.1	121.8	275.2	710.6
2015	244.2	193.8	311.9	223.2	147.8	270.1	248.9	206.6	120.3	268.8	700.4
2016	234.6	184.8	300.9	229	146.7	265.8	238.7	196.4	119	258.6	657.3
2017	227.5	177.4	293.7	205.7	147.2	251.1	232.1	193.3	116.5	250	634.8
2018	220.3	170.3	286.2	198.9	142.3	245.6	224.8	187.5	114.6	242	608
2019	209.6	161.3	272.9	196.8	132	234.3	213.7	183.1	109.6	230.5	574.8
2020	223.5	170.8	292.4	220.3	153.8	268.3	221.7	225.7	120.35	247.5	593
2021	233.5	178.7	304.9	203.1	157.2	262.9	238.4	210.9	122.63	255.16	642.86
2022	209.8	158.3	278	189.1	140.2	232.4	216	181.1	113.23	232.91	554.27
**Number of Joinpoints (years of Joinpoint)**	1 (2010)	1 (2011)	1 (2010)	-	1 (2014)	1 (2012)	1 (2010)	1 (2012)	1 (2010)	1 (2010)	1 (2012)
**APC-Segment 1 (95% CI)**	-6.03 (-6.38 to -5.75)	-6.03 (-6.42 to -5.77)	-6.12 (-6.48 to -5.83)	-4.39 (-4.82 to -3.97)	-5.52 (-6.28 to -5.16)	-5.84 (-6.66 to -5.51)	-5.95 (-6.29 to -5.69)	-6.07 (-6.78 to -5.61)	-6.40 (-6.79 to -6.11)	-6.18 (-6.47 to -5.95)	-5.55 (-7.22 to -5.13)
**APC-Segment 2 (95% CI)**	-3.63 (-4.07 to -3.02)	-4.09 (-4.67 to -3.11)	-3.24 (-3.68 to -2.65)	-	-2.48 (-4.14 to 1.71)	-3.93 (-4.93 to -1.06)	-3.44 (-3.87 to -2.85)	-3.13 (-4.22 to -1.04)	-2.10 (-2.57 to -1.49)	-3.61 (-4.02 to -3.09)	-4.45 (-5.32 to -1.17)
**Average APC**	-4.96	-5.26	-4.83	-4.39	-4.77	-5.18	-4.83	-5.05	-4.49	-5.03	-5.16

Supplemental Table 3. AMI-related mortality rates are stratified by the census region and urbanization status in the United States.

Year	Northeast	Midwest	South	West	Urban	Rural
1999	550.8	603.3	607	448.6	536.7	674.8
2000	533.4	576.3	591.6	428.1	516.7	656.1
2001	509.9	538	562	414.6	492.2	617.1
2002	489.3	519.6	541.4	406.4	473.4	605
2003	460.4	479.3	505.7	383.7	442.2	565.3
2004	416.4	430.5	460.7	352	400.3	517.9
2005	394.5	412.3	439.6	330.3	378.2	503.9
2006	360	388.3	398	308.2	347.4	466.9
2007	337.2	356.1	374.7	285.8	323.5	437.6
2008	327.6	352.2	365.5	280.8	313.9	441.3
2009	297	322.2	338.6	253.5	285.8	412.1
2010	284.4	307.4	324.2	245.3	274.4	393.1
2011	272.2	304.1	302.5	235.9	261.2	382.8
2012	257.6	290.6	289.5	225.9	247.9	372.3
2013	252.5	278.1	281	217.5	239.8	361.8
2014	237.8	269.4	267.8	202.2	226.4	351.5
2015	231.4	261.1	261.8	207	221.8	349.7
2016	219.6	250.8	250.8	202.6	213.1	337.2
2017	206	243	246.5	196.7	207.3	324.6
2018	200.4	237.3	239.2	187.2	200.3	317.6
2019	186.5	225.4	227.2	182.6	190	305.6
2020	196.1	237.6	245.5	194.3	202.6	326
2021	192.8	248.7	260.4	206.9	-	-
2022	173	225.5	230.8	189.5	-	-
**Number of Joinpoints (years of Joinpoint)**	1 (2010)	1 (2009)	1 (2011)	1 (2012)	1 (2011)	1 (2009)
**APC-Segment 1 (95% CI)**	-6.13 (-7.07 to –5.73)	-6.23 (-6.58 to -5.94)	-5.95 (-6.34 to -5.67)	-5.51 (-5.97 to -5.18)	-6.21 (-6.54 to -5.94)	-5.11 (-5.62 to -4.75)
**APC-Segment 2 (95% CI)**	-4.42 (-5.14 to -2.39)	-3.43 (-3.78 to -3.01)	-3.40 (-4.02 to -2.49)	-2.62 (-3.54 to -1.14)	-3.61 (-4.19 to -2.83)	-2.75 (-3.18 to -2.14)
**Average APC**	-5.36	-4.84	-4.94	-4.51	-5.18	-3.93

Supplemental Table 4. AMI-related change in AAMR at state level in United States.

State	Change in AAMR from 1999 to 2019	Change in AAMR from 2019 to 2020-2021
Alabama	-440.8	18.8
Alaska	-129.5	2.1
Arizona	-283	31.8
Arkansas	-255.2	-44

California	-295	24.1
Colorado	-240	0.9
Connecticut	-269.6	-5
Delaware	-445.3	12.8
District of Columbia	-261.6	1.2
Florida	-306.9	29.3
Georgia	-339	10.7
Hawaii	-205.8	8.8
Idaho	-331.4	38.8
Illinois	-446.3	10.8
Indiana	-360.1	34.2
Iowa	-339.4	0.2
Kansas	-291.6	8.2
Kentucky	-444.2	29.6
Louisiana	-443.6	29.7
Maine	-315.1	26
Maryland	-438.7	18.9
Massachusetts	-334.7	3.8
Michigan	-406.4	26.8
Minnesota	-261.9	11.1
Mississippi	-301.4	79.7
Missouri	-455.7	7.2
Montana	-253.5	43.1
Nebraska	-187.2	6.4
Nevada	-237.6	1.3
New Hampshire	-305.4	9
New Jersey	-433.5	6
New Mexico	-80.9	-74.6
New York	-333.3	2.7
North Carolina	-351.7	15.9
North Dakota	-398	-13.5
Ohio	-386.6	27.6
Oklahoma	-392.7	12.6
Oregon	-203.4	19.2
Pennsylvania	-404.6	21
Rhode Island	-417.2	-10.4
South Carolina	-440.7	35.5
South Dakota	-251.1	12.8
Tennessee	-418.3	44.7
Texas	-459.3	32.5
Utah	-261.1	21.2
Vermont	-342.3	9.2

Virginia	-300.4	26.1
Washington	-222.5	11.6
West Virginia	-416.4	19.6
Wisconsin	-353.4	17.5
Wyoming	-207.7	1
All US	23.9	18.7

Supplemental Table 5. Sensitivity analysis using AMI as an underlying cause of death, AMI + COVID and AMI – COVID deaths, and AAMR

Year	All AMI-related deaths	AMI as underlying cause of death (sensitivity analysis)	All AMI-related deaths (AAMR)	AMI as underlying cause of death (sensitivity analysis) (AAMR)	AMI + COVID 19 deaths	AMI deaths without associated COVID-19 (AMI -COVID-19)	AMI + COVID 19 (AAMR)	AMI deaths without associated COVID-19 (AMI -COVID-19) (AAMR)
1999	194044	163449	563.2	474.6	-	-	-	-
2000	189291	157414	543.5	452.1	-	-	-	-
2001	182116	150361	516	426	-	-	-	-
2002	177669	145311	498.4	407.6	-	-	-	-
2003	168226	136765	465.5	378.3	-	-	-	-
2004	154439	124616	422.5	340.7	-	-	-	-
2005	149410	119164	401.7	320.1	-	-	-	-
2006	140028	110158	369.7	290.5	-	-	-	-
2007	133128	102781	344.6	265.7	-	-	-	-
2008	133113	103545	337.4	262	-	-	-	-
2009	124226	96349	308.9	239.1	-	-	-	-
2010	121035	93040	296	227.1	-	-	-	-
2011	119111	91094	283.1	216.1	-	-	-	-
2012	117083	89431	270.1	206	-	-	-	-
2013	116479	88742	261.5	198.9	-	-	-	-
2014	113582	86266	248.5	188.4	-	-	-	-
2015	114613	86160	244.2	183.3	-	-	-	-
2016	112837	84409	234.6	175.3	-	-	-	-
2017	112402	83373	227.5	168.5	-	-	-	-
2018	111987	82240	220.3	161.8	-	-	-	-
2019	109282	79132	209.6	151.8	-	-	-	-
2020	119373	81857	223.5	153.2	6442	112931	12.1	211.4
2021	121052	81551	233.5	157.6	8471	112581	16.3	217.2
2022	115016	78722	209.8	143.7	6303	108713	11.6	198.2

Supplemental Figure 1. APC for overall and gender stratified AMI-related mortality rate in the older adult population of US United States from 1999 to 2019. *Indicates the APC is significantly different from 0.

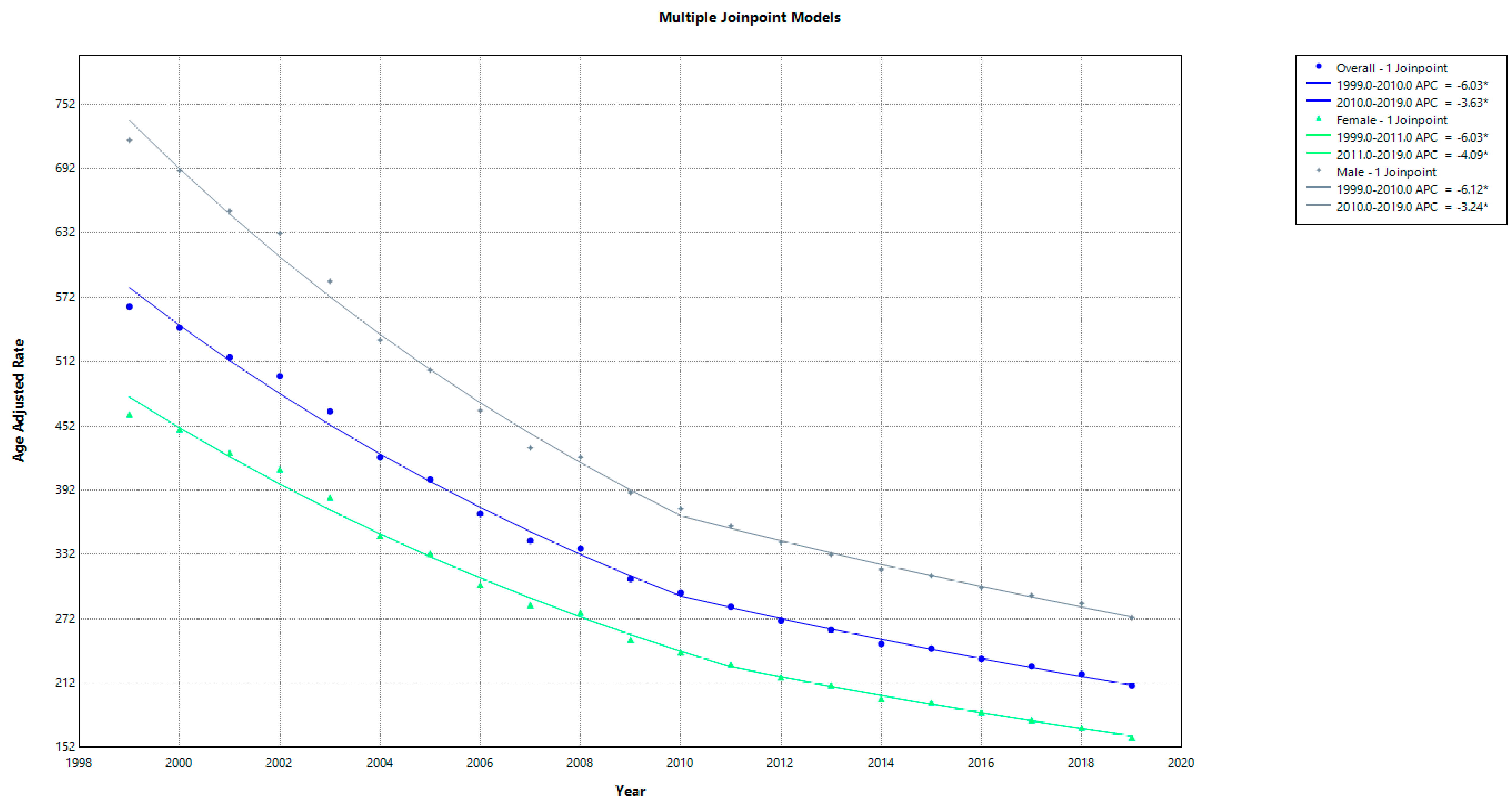

Supplemental Figure 2. APC for Race stratified AMI-related mortality rate in the older adult population of US United States from 1999 to 2019. *Indicates the APC is significantly different from 0.

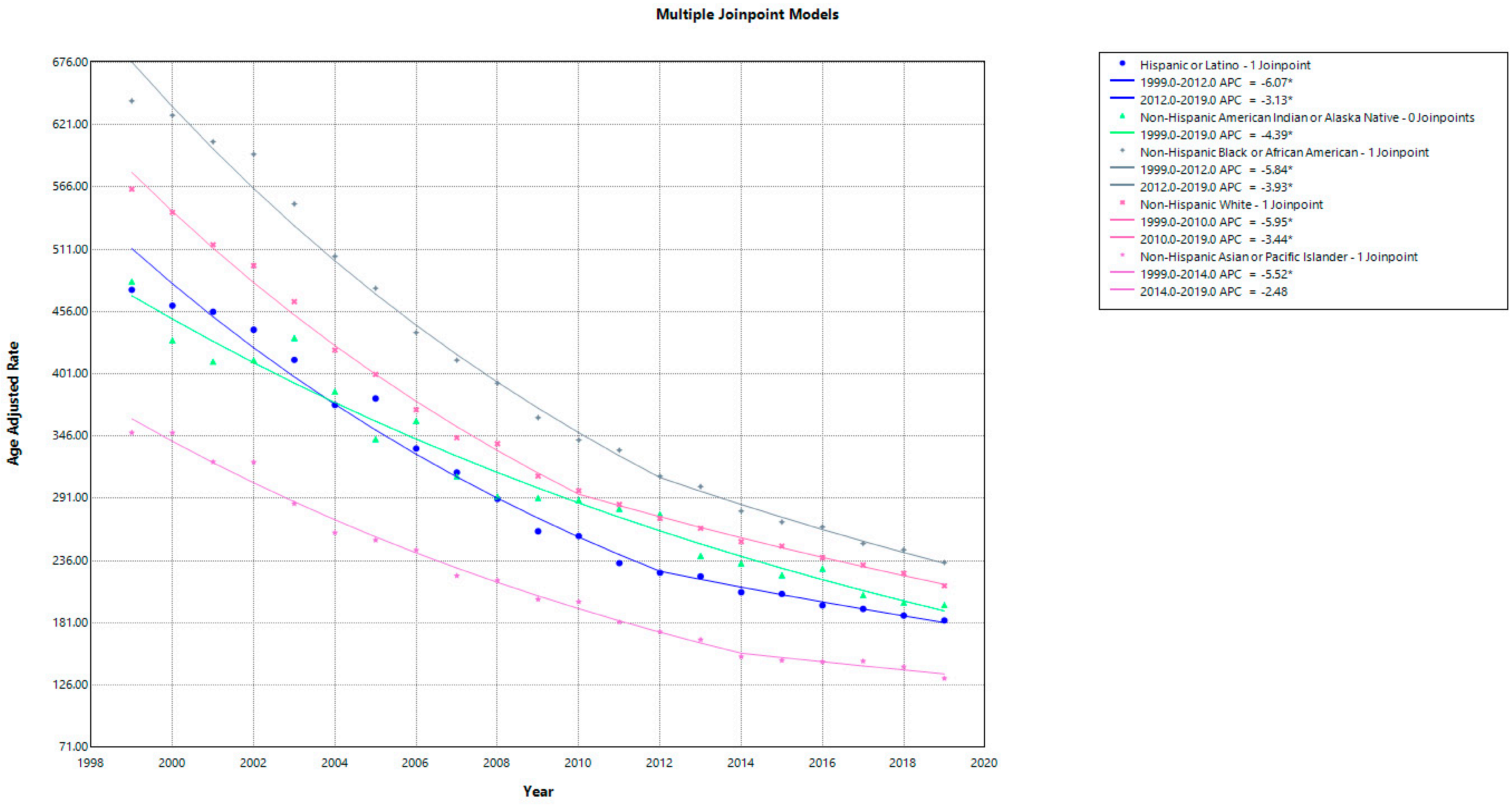
Multiple Joinpoint Models
676.00
621.00
566.00
511.00
456.00
401.00
346.00
291.00
236.00
181.00
126.00
71.00
Age Adjusted Rate
1998
2000
2002
2004
2006
2008
2010
2012
2014
2016
2018
2020
Year
Hispanic or Latino - 1 Joinpoint
1999.0-2012.0 APC = -6.07*
2012.0-2019.0 APC = -3.13*
Non-Hispanic American Indian or Alaska Native - 0 Joinpoints
1999.0-2019.0 APC = -4.39*
Non-Hispanic Black or African American - 1 Joinpoint
1999.0-2012.0 APC = -5.84*
2012.0-2019.0 APC = -3.93*
Non-Hispanic White - 1 Joinpoint
1999.0-2010.0 APC = -5.95*
2010.0-2019.0 APC = -3.44*
Non-Hispanic Asian or Pacific Islander - 1 Joinpoint
1999.0-2014.0 APC = -5.52*
2014.0-2019.0 APC = -2.48

Supplemental Figure 3. APC for age-stratified AMI-related mortality rate in the older adult population of the United States from 1999 to 2019. *Indicates the APC is significantly different from 0.

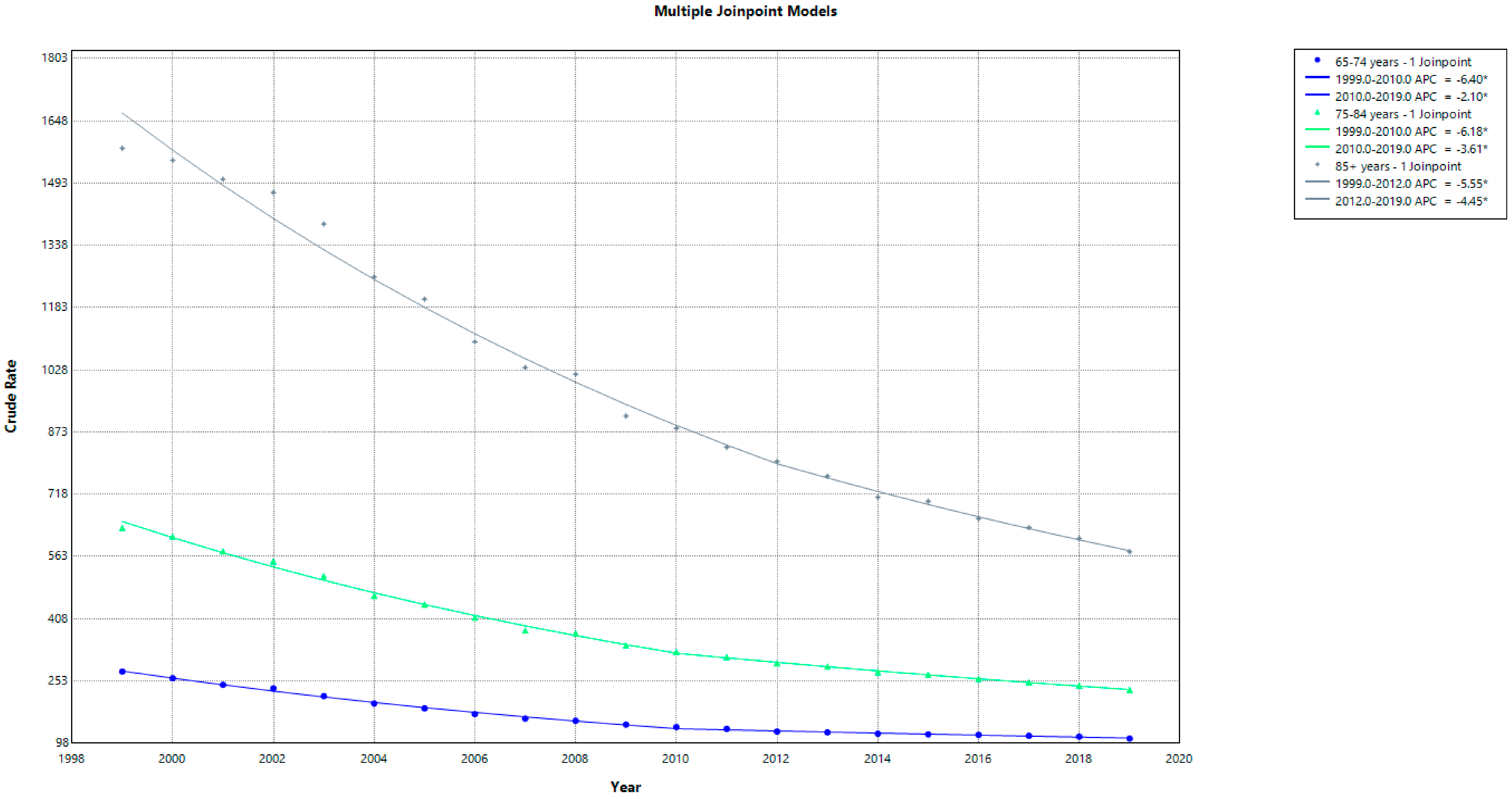

Supplemental Figure 4. APC for census region-stratified AMI-related mortality rate in the older adult population of the United States from 1999 to 2019. *Indicates the APC is significantly different from 0

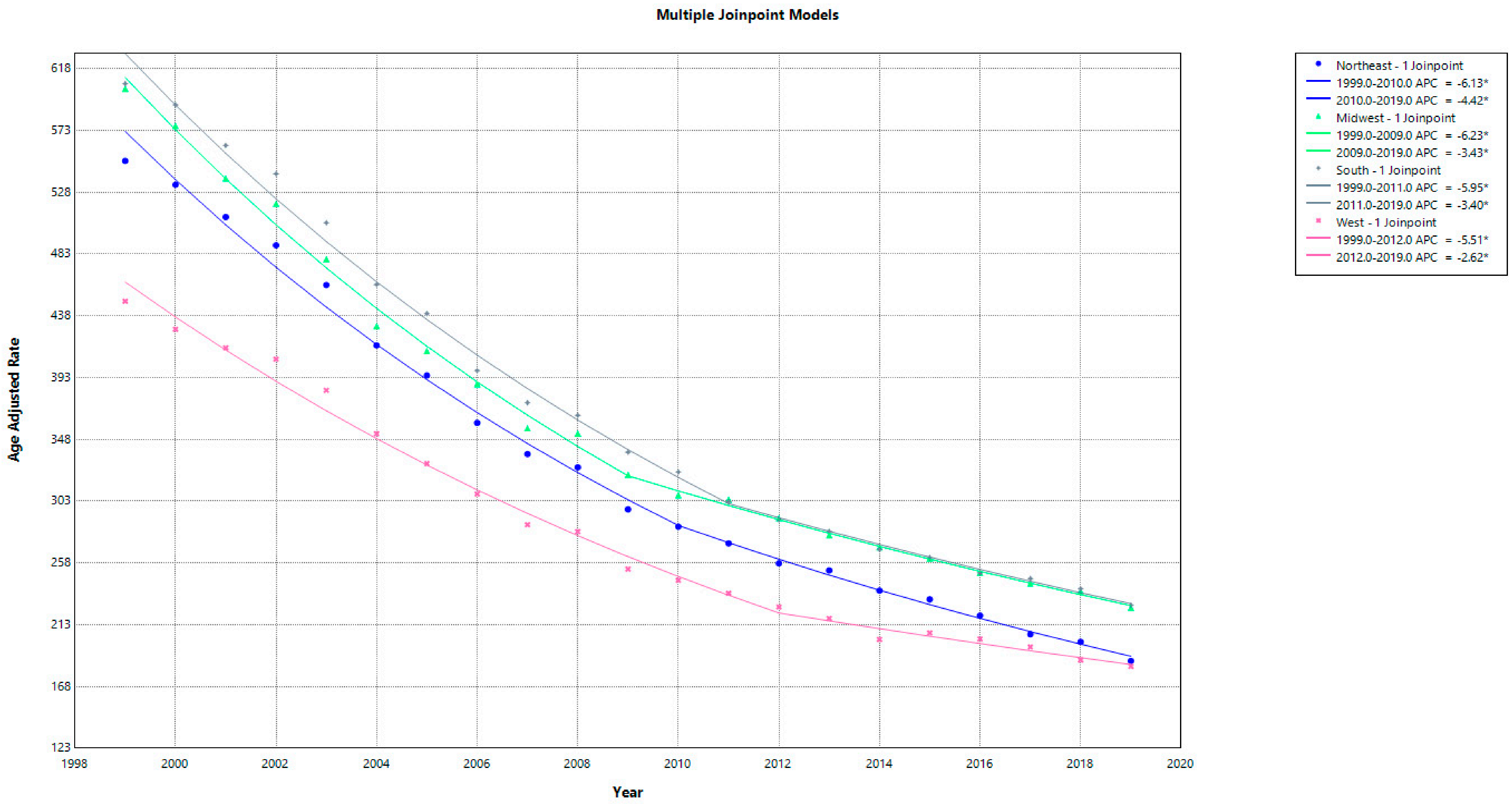

Supplemental Figure 5. APC for urban-rural-stratified AMI-related mortality rate in the older adult population of the United States from 1999 to 2019. *Indicates the APC is significantly different from 0

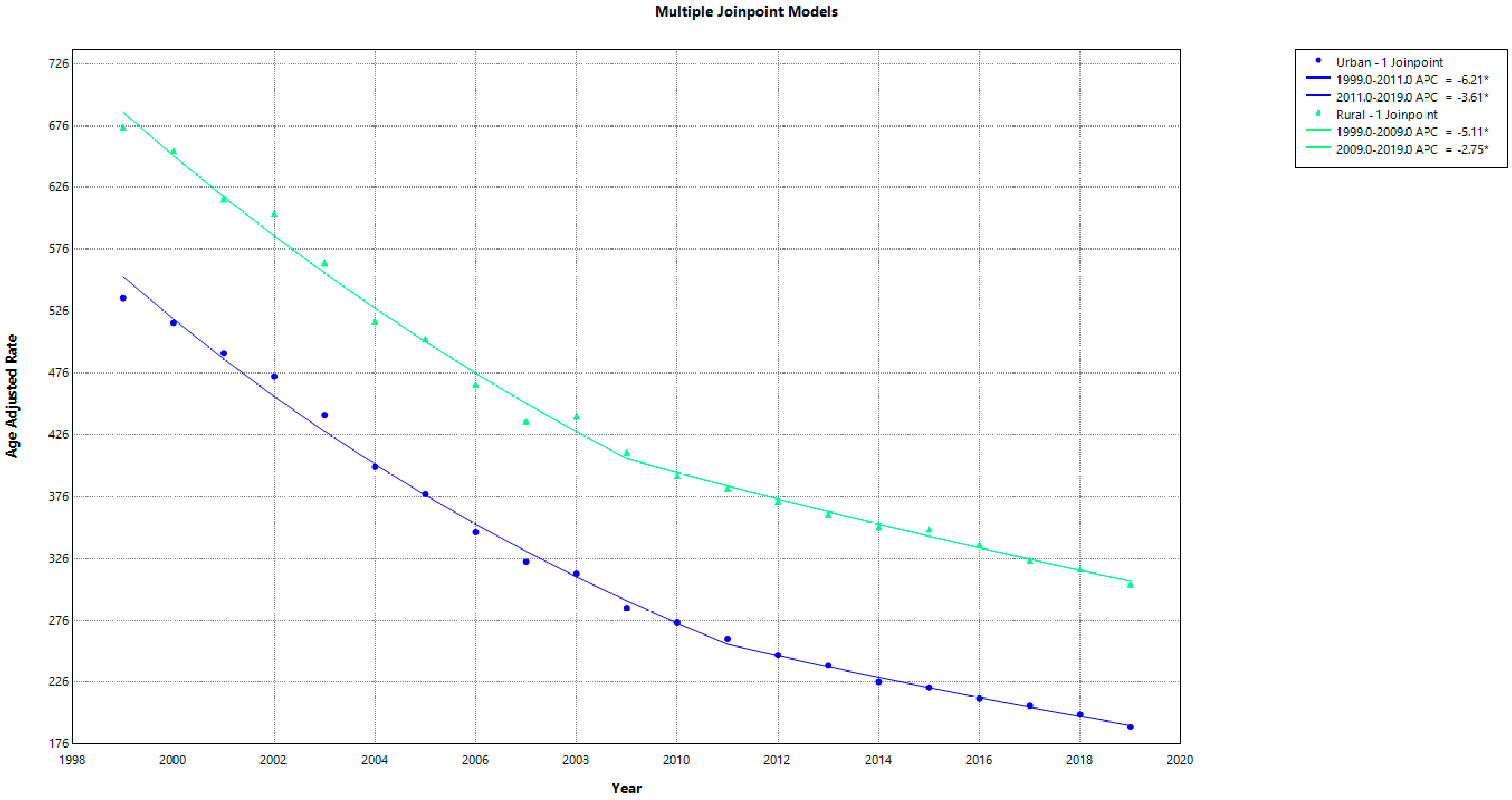

Supplemental Figure 6. Sensitivity analysis and AMI deaths without COVID-19

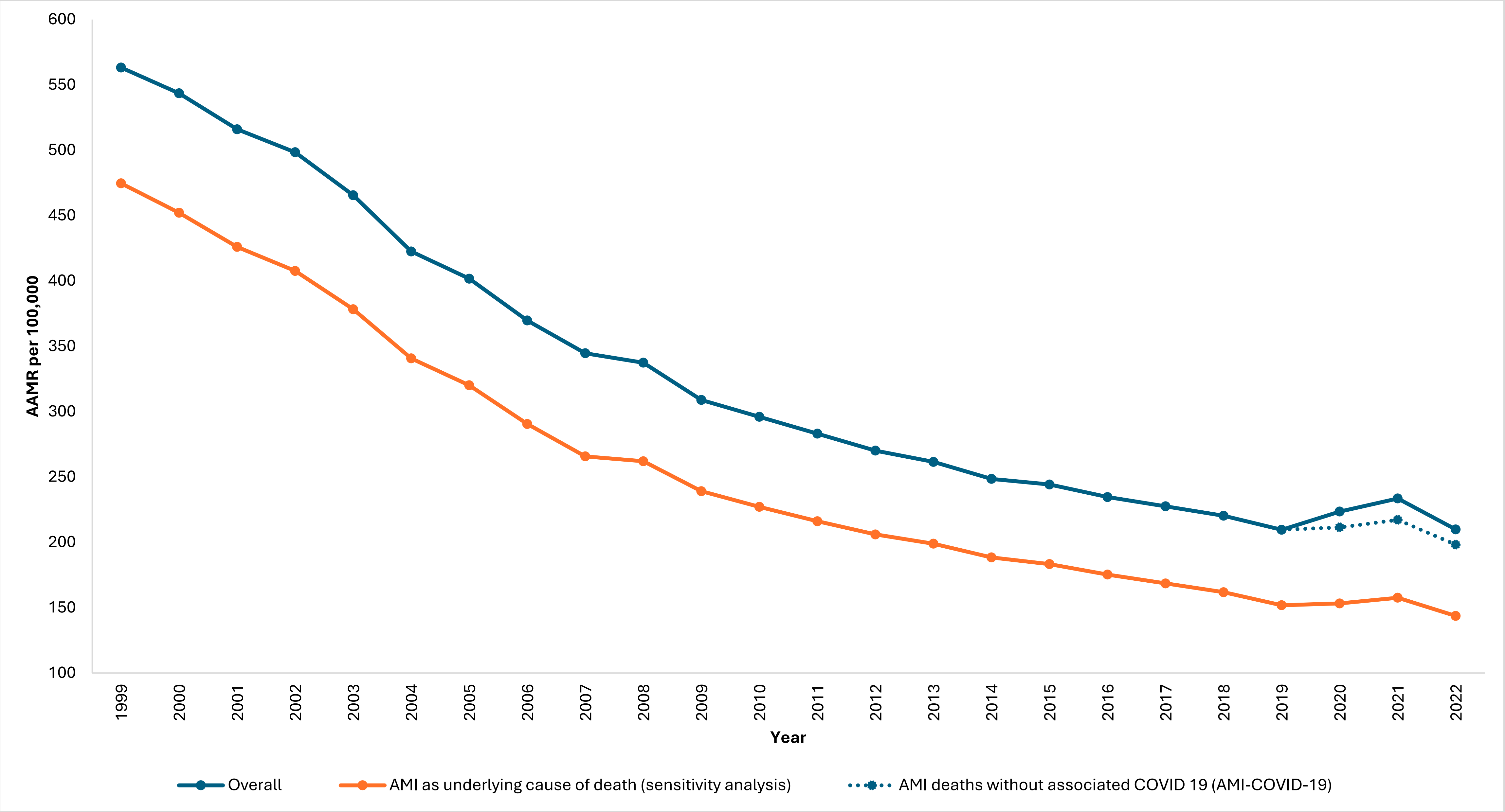

Supplemental Figure 7. State-level change in AMI-related AAMR from 1999 to 2019

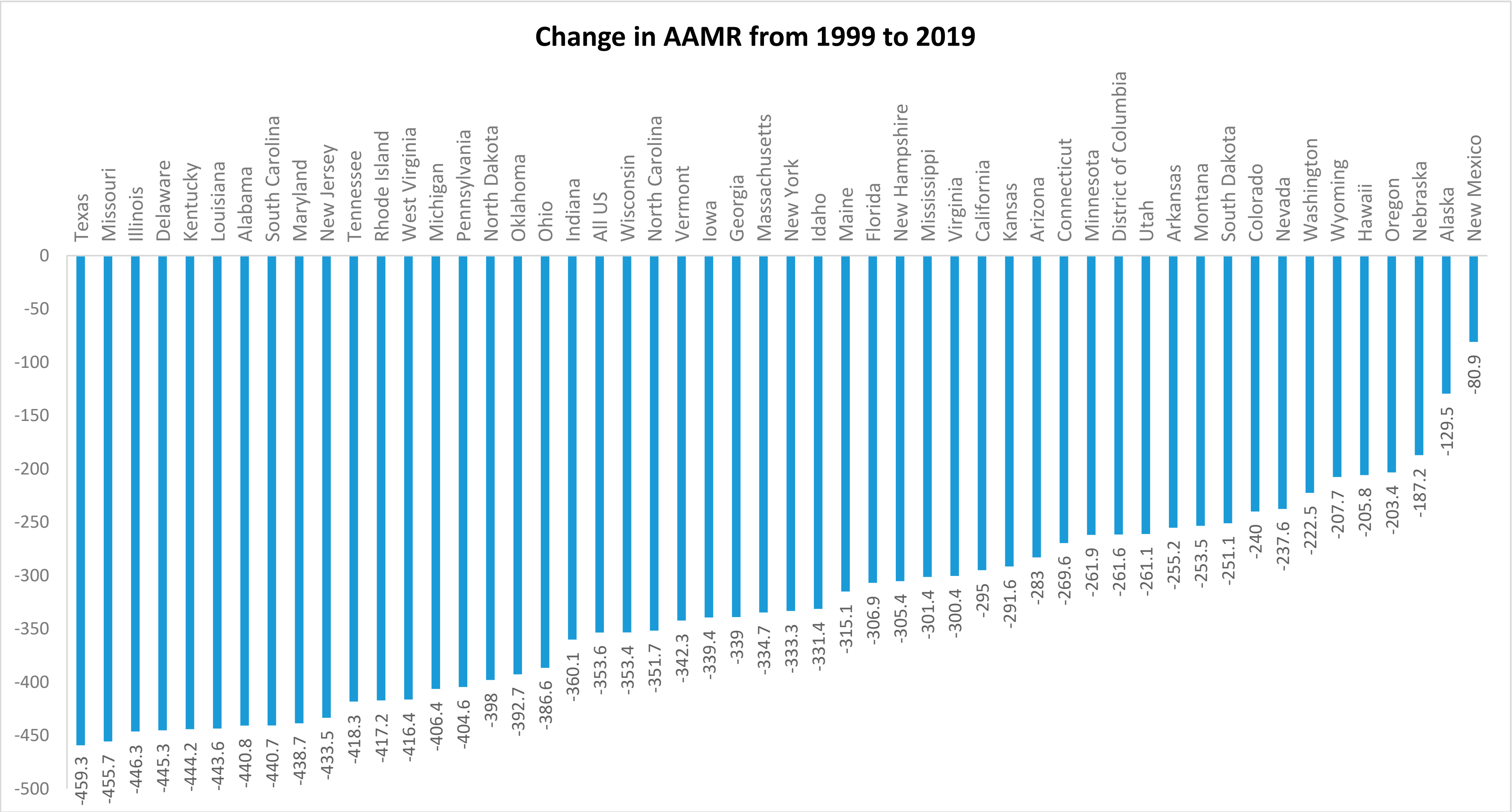

Supplemental Figure 8. State-level change in AMI-related AAMR from 2019 to 2020-2021 period

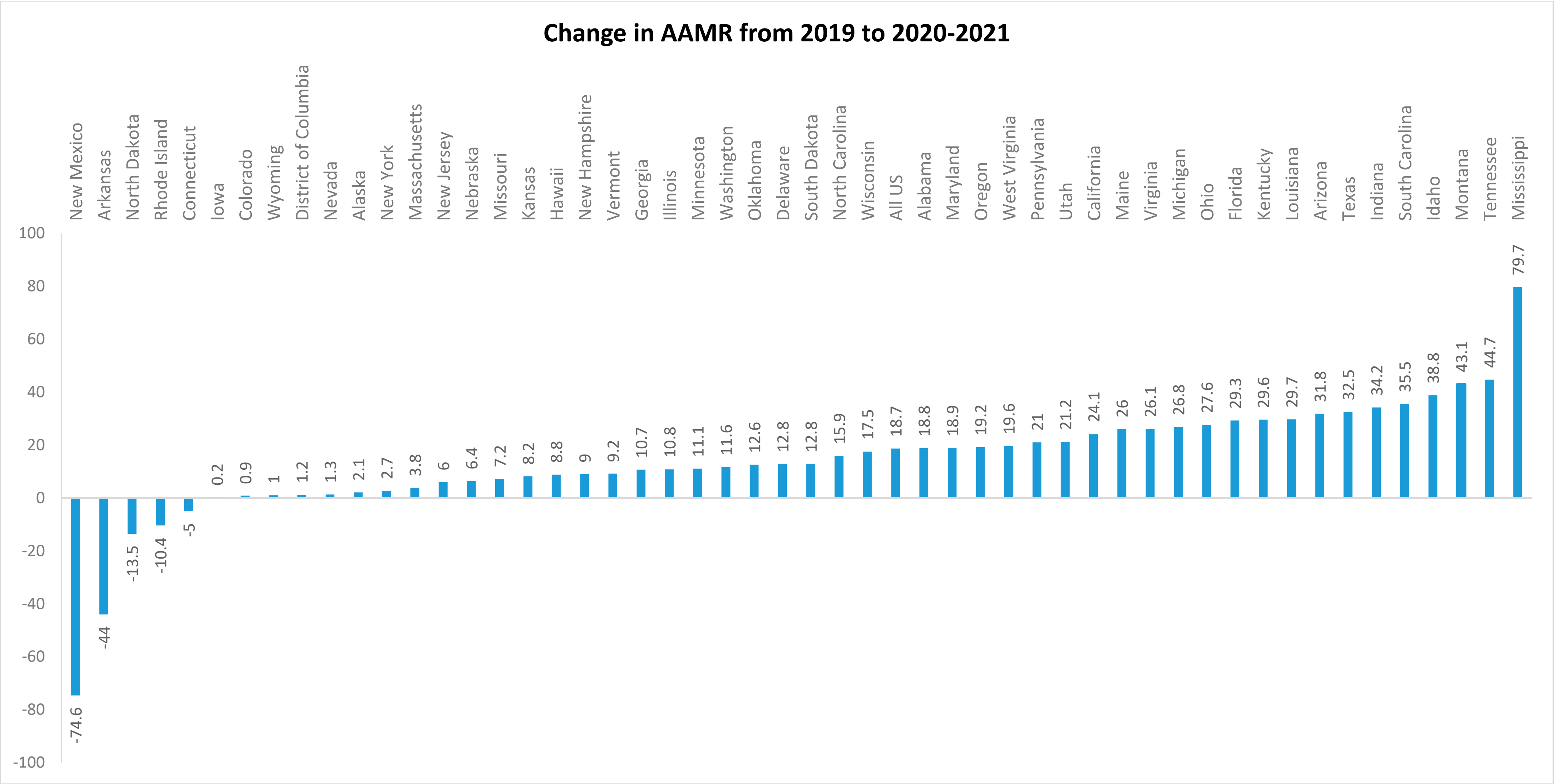

Supplement: Supplementary file 1 [file jcm-14-02190-s001.zip › jcm-3519139-supplementary.pdf]
